# Non-Enhanced T1-Weighted Liver Vessel Imaging at 7 Tesla

**DOI:** 10.1371/journal.pone.0097465

**Published:** 2014-06-02

**Authors:** Anja Fischer, Oliver Kraff, Stefan Maderwald, Karsten Beiderwellen, Mark E. Ladd, Michael Forsting, Thomas C. Lauenstein, Lale Umutlu

**Affiliations:** 1 Department of Diagnostic and Interventional Radiology and Neuroradiology, University Hospital Essen, Essen, Germany; 2 Erwin L. Hahn Institute for Magnetic Resonance Imaging, University Duisburg-Essen, Essen, Germany; 3 German Cancer Research Center (DKFZ), Heidelberg, Germany; University Medical Center (UMC) Utrecht, Netherlands

## Abstract

**Objectives:**

Aim of the study was to assess the feasibility and to compare three non-enhanced T1-weighted (w) sequences for liver vessel imaging at 7 Tesla (T).

**Material and Methods:**

12 healthy volunteers were examined on a 7 T whole-body MR-system. The following non-enhanced sequences were acquired: T1w 2D FLASH, T1w 3D FLASH and Time of flight (TOF)-MRA. Qualitative image analysis was performed by two radiologists including over all image quality as well as vessel delineation of the liver arteries, liver veins and portal vein and the presence of artifacts using a five-point scale (5 = excellent vessel delineation to 1 = non-diagnostic). Contrast ratios (CR), SNR und CNR of the above named vessels in correlation to adjacent liver tissue were calculated for quantitative assessment. For statistical analysis, a Wilcoxon Rank Test was applied.

**Results:**

All three sequences provided a homogenous hyperintense delineation of the assessed liver vessels. Qualitative image analysis demonstrated the superiority of TOF-MRA, providing best overall image quality (TOF 4.17, 2D FLASH 3.42, 3D FLASH 3.46; p<0.01) as well as highest image quality values for all analyzed liver vessel segments. TOF-MRA was least impaired by B_1_ inhomogeneity (4.13) and susceptibility artifacts (4.63) out of all three sequences (p<0.01). Quantitative image analysis confirmed the superiority of TOF MRA showing significant higher CR values for all liver vessels (e.g. right hepatic artery TOF 0.47, 2D FLASH 0.09, 3D FLASH 0.11 with p = 0.02 and 0.01, respectively). Providing the lowest standard deviation in noise, TOF showed highest values for SNR and CNR.

**Conclusions:**

Non-enhanced T1w imaging in general and TOF MRA in particular, appear to be promising techniques for high quality non-enhanced liver vessel assessment at 7 T.

## Introduction

Over the last decades, contrast-enhanced (ce) CT angiography and MR angiography (MRA) have been established as the imaging techniques of choice to assess liver vessel anatomy prior to hepatectomy or liver transplantation [1], [2]. As CT angiography deploys ionizing radiation and the application of contrast agent in general bears the potential of side effects and is restricted in case of renal insufficiency [3], the focus of scientific research has shifted towards non-enhanced MRA techniques within the last few years. Several studies at lower field strength (1.5 Tesla) have demonstrated the diagnostic potential of non-contrast-enhanced liver vessel imaging, utilizing true steady-state free-precession sequences and time-spatial labeling inversion pulses. Shimada et al. reported initial results of 3D true SSFP imaging utilizing a spatial labeling inversion pulse to visualize hepatic arterial and venous vasculature, selectively achieving high arterial and venous vessel contrast and good suppression of the portal veins and surrounding static tissue [4]–[6].

In clinical routine, the standard magnetic field strength for imaging of hepatic vessels lies in 1.5 T MRI. The increase of the magnetic field strength to 3 T has been proven beneficial in terms of achieving higher spatiotemporal resolution, as well as enabling comparable or improved delineation of vessel structures [7]. With recent development in multi-channel transmit/receive RF (radiofrequency) body coil technology, the feasibility of ultra-high field imaging (7 T) of abdominal organs and vessel structures could be demonstrated [8]–[10]. A successful transformation of the increased signal-to-noise ratio into imaging at high spatiotemporal resolution could be observed, displaying highly defined anatomical details of parenchymatous organs and arterial vasculature [11], [12]. Nevertheless, MR imaging at higher field strength also entails challenges, dealing with limitations based on increasing signal heterogeneities due to RF wavelength effects and restrictions due to increasing SAR (specific absorption rate) [13]–[15]. With SSFP and T2 TSE imaging being restricted at ultra-high-field strength, most studies on 7 Tesla non-enhanced MRA have focused on T1-weighted imaging, utilizing the inherently hyperintense vessel signal at 7 T. Thus, non-enhanced MRA at 7 T has been successfully demonstrated for imaging of the intracranial and renal vasculature as well as vessels of the lower extremities [16]–[19].

The aim of this study was to investigate the feasibility of non-enhanced MR imaging of the arterial, venous and portal liver vessels comparing three T1w sequences at 7 Tesla.

## Materials and Methods

### Study Population

12 healthy volunteers were enrolled in this trial, including seven female and five male subjects. The average age was 31.2 years with a range of 23 to 44 years. The study was conducted in conformance with the Declaration of Helsinki and approved by the Ethics Commission of the Medical Faculty of the University Duisburg-Essen (study number 11-4898-BO). Written informed consent was obtained from each volunteer before the examination. After leaving the examination room all subjects were consulted about possible side effects.

### Scanner and Coil System

**Figure 1:**
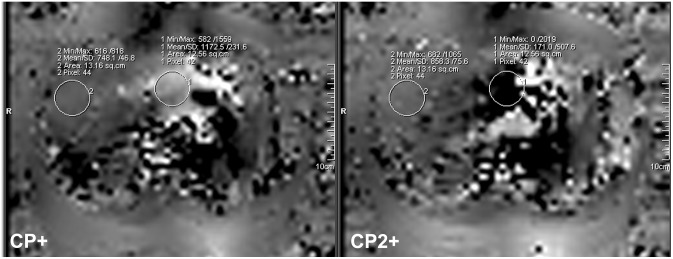
Flip angle maps obtained in two standard excitation modes in transversal orientation of the upper abdomen. Improved excitation of the liver is given with the CP^2+^ mode compared to the CP^+^ mode both in terms of general coverage, particularly of the right liver lobe as well as regarding mean reached flip angle. In the images, ROIs are displayed ventral of the aorta (ROI1) and within liver tissue of the right liver lobe (ROI2) yielding flip angle values encoded by a factor of 10.

Examinations were performed on a 7 Tesla whole-body MR system (Magnetom 7T, Siemens Healthcare Sector, Erlangen, Germany) in supine position. For image acquisition a custom-built eight channel body transmit/receive RF coil was utilized [20]. The body coil consisted of two arrays with four elements each placed ventral and dorsal on the upper abdomen. The field of view (FOV) was centered on the liver. In this region, manual adjustments, in particular B_0_ shimming was performed utilizing a shim volume adapted to the region of interest. As MRI at higher field strength is known to be susceptible to transmit B_1_ field inhomogeneities due to the reduced Larmor wavelength [21], static shimming of the eight individual transmit phases of the RF coil was performed to mitigate potential limitations and to achieve a more homogeneous excitation of the upper abdomen [22]. A phase increment of 90 degrees and equal amplitudes between neighboring elements (circularly polarized mode CP^2+^) was found to be most compatible for this study in terms of image quality as it constrained the signal voids to a periaortal focus as shown in Figure 1. Here, flip angle maps obtained in transversal orientation with the Bloch-Siegert-Shift B1 mapping technique [23], [24] demonstrate advantages of the CP^2+^ mode for the study objective in comparison to the CP^+^ mode. Furthermore, predefined phase settings allow an improved workflow as input power limits can be calculated prior to the study for the SAR supervision. For compliance with the International Electrotechnical Commission (IEC) guidelines [25], SAR calculations were performed in human adult male and female body models of the Virtual Family (CST Microwave Studio, Darmstadt, Germany) [26] prior to the study, resulting in a maximum permitted input power level of 24 W for which the localized 10 g-averaged SAR complies with the limit of 10 W/kg for both human body models used in the simulations. Given is the total RF power at the coil plug, which was equally split to all eight coil elements.

### Sequence Protocol

With the increase of the magnetic field strength, sequence parameters need adjustments and modifications to provide best possible image quality while providing a good workflow for the whole imaging protocol and obeying RF power constraints. Therefore imaging parameters (e.g. repetition time (TR), echo time (TE), flip angle (α) and bandwidth) were adapted and modified to 7 Tesla in an exclusive group of three healthy volunteers prior to the start of the study. The examination protocol consisted of the following sequences:

1. T1w spoiled gradient-echo sequence (2D FLASH) in transversal orientation.
2. T1w spoiled gradient-echo sequence (3D FLASH) in transversal orientation.
3. T1w 2D FLASH Time-of-Flight (TOF) MRA in transversal orientation.

Aim of the optimization process was to achieve high contrast between vasculature and parenchyma at a high spatial resolution for the delineation of the vessels. However, nominal flip angles and transmit reference voltages in the sequences were set to allow continuous scanning without waiting times between individual scans to allow the subjects to cool down in case SAR limits would have been reached within the 6-minute averaging time-window requested by the safety guidelines. Achieving an acquisition time within breath-hold conditions for the rather high flip angle TOF sequence was found difficult. Best results regarding image quality and workflow were obtained by using a lower spatial resolution compared to FLASH acquisitions. However, to compensate for the reduced matrix the FOV of the TOF sequence was reduced to a minimum for whole-liver coverage. The above mentioned SAR limitations in the TOF sequence were also the rationale for including the other two gradient echo sequences in this study. Prior publications have demonstrated the feasibility of non-contrast enhanced MRA of those standard gradient echo sequences for intracranial or renal vessel imaging at 7 T [12], [27]. Furthermore, it has to be mentioned, that 2D and 3D FLASH imaging were acquired at a higher spatial and temporal resolution which might be beneficial for a high quality vessel delineation and for clinical applicability providing a faster image acquisition. Imaging parameters of the above named sequences are summarized in Table 1. The total examination time was registered using a stop watch.

**Table 1:** Sequence parameters.

	2D FLASH	3D FLASH	TOF
**Repetition time TR [ms]**	193	3.2	17
**Echo time TE [ms]**	3.57	1.36	4.7
**Nominal Flip angle FA [°]**	70	10	60
**Bandwidth [Hz/pixel]**	410	930	230
**Slice orientation**	transversal	transversal	transversal
**Slices**	42	104	20
**Slice thickness [mm]**	5	2	2.5
**Field-of-view FOV [mm^2^]**	350	350	230
**Acquisition matrix [pixel]**	448×448	448×448	256×128
**Parallel imaging with GRAPPA**	Yes (R = 2)	Yes (R = 2)	Yes (R = 2)
**Reference lines**	48	48	48
**Acquisition time TA [min∶s]**	0∶52(breathhold with 2 concatenations)	0∶24	0∶33(breathhold with 5 concatenations)

### Data Analysis

All images were analyzed separately and blinded by two radiologists with 8 and 3 years experience in abdominal MRI on a standard Picture Archiving and Communication System (PACS) workstation (Centricity RIS 4.0i, GE Healthcare, USA). Qualitative image analysis was performed in the transversal source images for following vessel segments:

1. *arterial*
  - proper hepatic artery (PHA)
  - 2^nd^ order branch of the right hepatic artery in liver segment 5 (RHA)
2. *portal venous*
  - main portal vein (PV)
  - 1^st^ order branch of the right portal vein (RPV)
3. *venous*
  - inferior vena cava (IVC)
  - middle hepatic vein (MHV).

Visual qualitative image analysis was carried out with regard to vessel delineation using a five-point scale (5 = excellent image quality with sharply defined vessels, 4 = good vessel delineation, 3 = moderate vessel delineation, 2 = poor vessel delineation, 1 = not visible or non-diagnostic) as well as overall image quality for each sequence. Additionally presence of artefacts was assessed for each sequence using five categories: 1 = massive impairment, non-diagnostic, 2 = strong impairment, 3 = moderate impairment, 4 = slight impairment or insignificant, 5 = no artifact present. The following artefacts were evaluated: (1) B_1_ inhomogeneity, (2) inflow effects, (3) motion artefacts, (4) susceptibility artefact and (5) overall image impairment.

For quantitative image evaluation, contrast ratios (CR = [S_vessel_−S_liver_]/[S_vessel_+S_liver_]) of the above named vessel segments were assessed by placing ROIs (regions of interest) of identical size in the according vessel lumen and neighboring liver tissue.

Measurements of signal-to-noise and contrast-to-noise ratio (SNR, CNR) were performed for all vessel segments: [SNR_vessel_ = Signal_vessel_/noise], [SNR_liver_ = Signal_liver_/noise], [CNR = SNR_vessel_−SNR_liver_]. To accommodate for inhomogeneous noise due to the use of parallel imaging[28], it was measured by placing two maximum size ROIs out of the body and to use the mean value of the standard deviation of each noise value.

For statistical analysis, a Wilcoxon Rank Test was applied (Software: IBM SPSS Statistics 21). Score values for image quality and presence of artifacts were compared for each sequence. A p-value<0.05 was considered to establish statistically significant differences. In addition, interobserver reliability was calculated using the method of Landis et al. [29].

## Results

All MR examinations at 7 T were successfully performed and well tolerated by the subjects without any side effects. The examination time amounted to 16 min (+/−2 min) including positioning, manual adjustments and data acquisition of the non-enhanced sequences.

**Figure 2:**
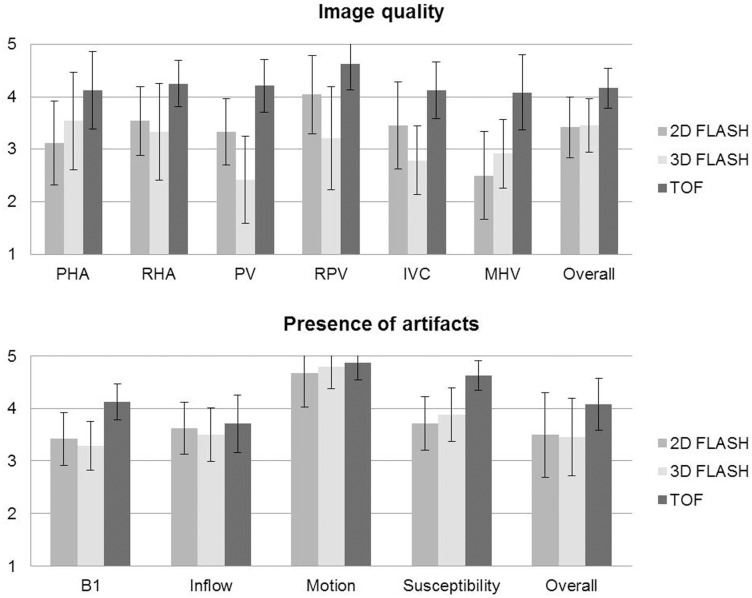
Qualitative analysis of image quality and presence of artifacts (5-point scales) for all analyzed liver vessel segments in all three sequences. (PHA  =  proper hepatic artery, RHA = 2^nd^ order branch of right hepatic artery, PV  =  main portal vein, RPV = 1^st^ order branch of right portal vein, IVC  =  inferior vena cava, MHV  =  middle hepatic vein).

Qualitative image analysis showed the superiority of TOF MRA, which provided best overall image quality (4.17±0.38) as well as highest image quality values for all analyzed liver vessel segments and was least impaired by artifacts (overall artifacts 4.08±0.28) (Fig. 2). 2D FLASH and 3D FLASH images revealed similar values, in terms of moderate to good overall image quality ratings (2D FLASH 3.42±0.58, 3D FLASH 3.46±0.51) and a low to moderate presence of artifacts (overall 2D FLASH 3.50±0.51, 3D FLASH 3.46±0.51). Nevertheless among the two sequences, 2D FLASH MRI yielded superior conspicuity of all assessed liver vessels despite the common hepatic artery and the middle hepatic vein.

**Figure 3:**
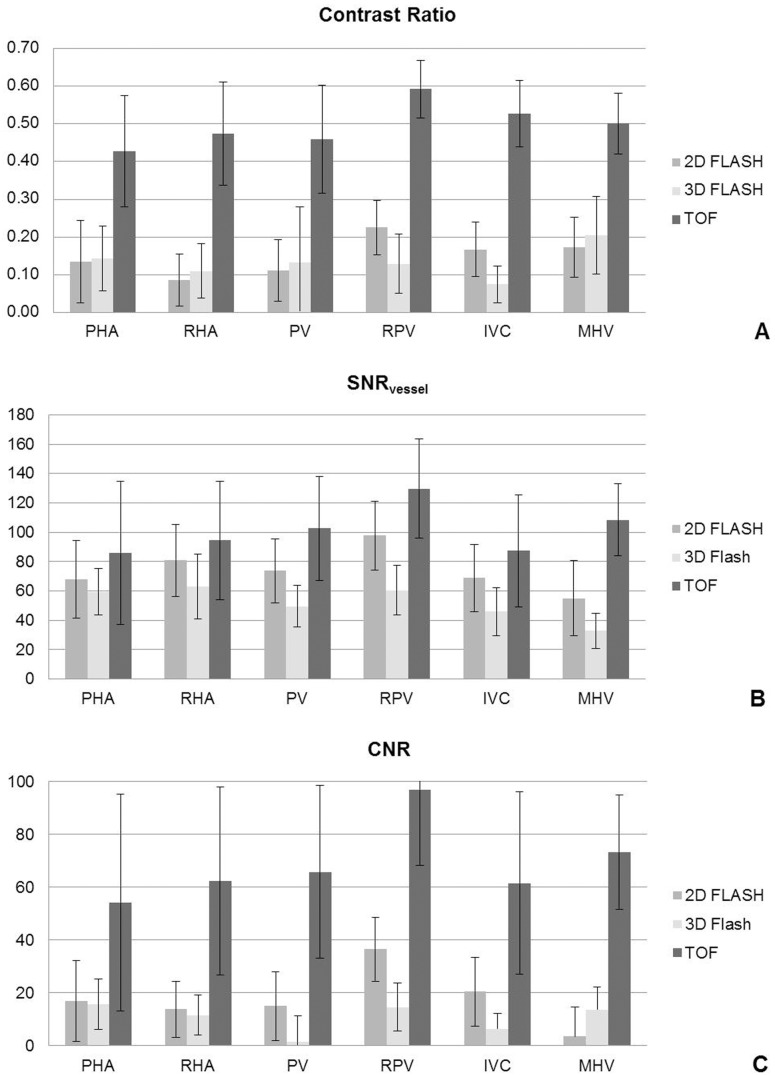
Quantitative analysis (A: Contrast ratios, B: SNR_vessel_, C: CNR) for all analyzed liver vessel segments in all three sequences.

Quantitative image analysis confirmed the results of the qualitative image evaluation, in terms of superiority of the TOF sequence. TOF MRA showed highest CR values for the right portal vein (0.59±0.08), closely followed by the inferior vena cava and the middle hepatic vein (0.53±0.09, 0.50±0.08, respectively). In contrast, 2D and 3D FLASH imaging, achieved a considerably lower vessel contrast for all analyzed segments with values ranging from 0.07±0.05 (IVC at 3D FLASH) to 0.23±0.07 (RPV at 2D FLASH). TOF MRA provided comparable superiority for SNR and CNR measurements, yielding highest scores for SNR and CNR in all vessel segments (highest SNR and CNR for RPV: TOF SNR 129.73±33.98 and CNR 96.94±28.74, 2D FLASH SNR 97.75±23.33 and CNR 36.39±12.22, 3D FLASH SNR 60.44±16.88 and CNR 14.28±9.33). Mean scores for the qualitative analysis and the presence of artifacts are displayed in Figure 2. Figure 3 shows the mean CR, SNR and CNR values for all sequences. Detailed results for each sequence are described below.

### T1w Spoiled Gradient-echo Sequence (2D FLASH)

**Figure 4:**
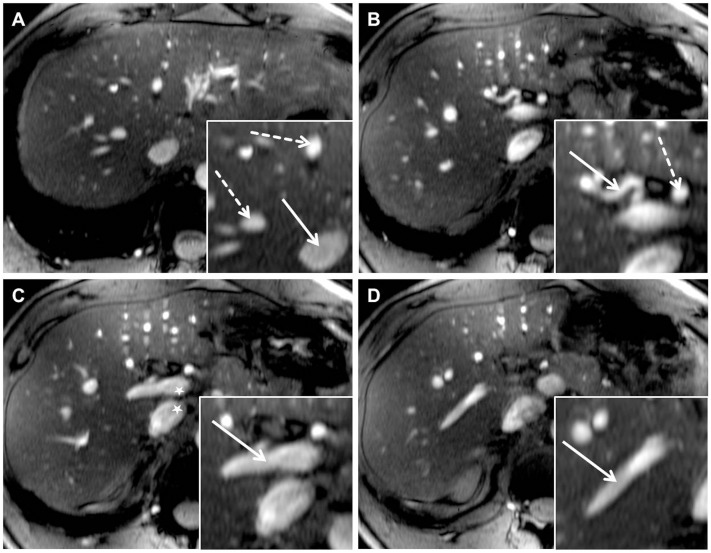
TOF MRA demonstrated high quality delineation of all analyzed liver vessel segments due to a hyperintensive vessel signal and an effective suppression of background signal. Figure A shows the inferior vena cava (arrow) as well as the right and middle hepatic veins (dashed arrow). A major part of the right hepatic artery (arrow) and a section of the common hepatic artery (dashed arrow) are visualized in Figure B. Figure C and D show the hyperintense delineation of the main portal vein (arrow) and the right portal vein (arrow). A slight intraluminal signal inhomogeneity could be depicted in the main portal vein and inferior vena cava (Figure C, star). Furthermore, signal drop outs due to susceptibility are visible in the stomach in the left ventral abdomen, but do not hamper vessel visualization (Figure B–D).

Qualitative analysis of 2D FLASH imaging demonstrated a moderate delineation of the hepatic arteries (PHA 3.13±0.80, RHA 3.54±0.66) and veins (IVC 3.46±0.83, MHV 2.50±0.83). The sequence was moderately impaired by B_1_ (3.42±0.50) and inflow (3.63±0.49) artifacts, mainly located in the periaortal region (Fig. 4). Signal loss due to susceptibility and motion artifacts caused only a slight disturbance of vessel delineation (mean values 3.71±0.81, 4.67±0.64, respectively).

Regarding quantitative evaluation, 2D FLASH imaging provided CR values ranging from 0.09 (RHA±0.07) to 0.23 (RPV±0.07), which were significantly lower compared to TOF imaging (RHA p = 0.02, RPV p = 0.02), yet marginally higher than for 3D FLASH MRI.

Assessment of interobserver reliability revealed a moderate agreement with a mean kappa value of 0.51 for qualitative analysis of all liver vessels. Regarding the presence of artifacts, a slightly higher strength of agreement was detected (mean 0.58).

### T1w Spoiled Gradient-echo Sequence (3D FLASH)

Out of all three sequences, 3D FLASH MRI was the sequence to provide poorest vessel delineation, particularly for assessment of the main portal vein (2.42±0.83), inferior vena cava (2.79±0.66) and the middle hepatic vein (2.92±0.65) with statistically significant difference to TOF MRA (p-values ranging from to 0.00 to 0.012). Similar to 2D FLASH imaging, there was a moderate impairment caused by B_1_ (3.29±0.46) and inflow artifacts (3.50±0.51) as well as a minor presence of susceptibility artifacts (3.88±0.74).

Quantitative analysis confirmed the predominantly moderate to poor vessel delineation at 3D FLASH imaging: CR values ranged from 0.07 (IVC±0.05) to 0.20 (MHV±0.10), being significantly lower compared to TOF imaging (IVC p = 0.02, MHV p = 0.02).

The interobserver agreement was rated moderate (mean 0.44) for vessel delineation and substantial (mean 0.63) for the presence of artifacts.

### T1w Time-Of-Flight Sequence (TOF-MRA)

**Figure 5:**
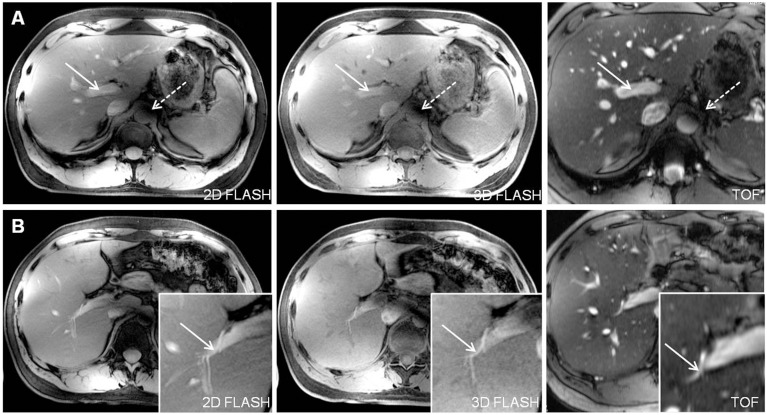
Depiction of the main portal vein (Figure A, arrows) and the right hepatic artery (Figure B, arrows) in all three sequences in one subject. 2D and 3D FLASH provided moderate to good image quality for both vessel segments with a slight advantage for 2D FLASH imaging due to a marginally higher contrast. TOF MRA was superior in the delineation of the liver arteries and veins from proximal to peripheral segments. Signal voids due to remaining B_1_-inhomogeneity were shifted out of the liver to a periaortal focus, causing minor impairment of vessel delineation (dashed arrow in Fig. A).

**Figure 6:**
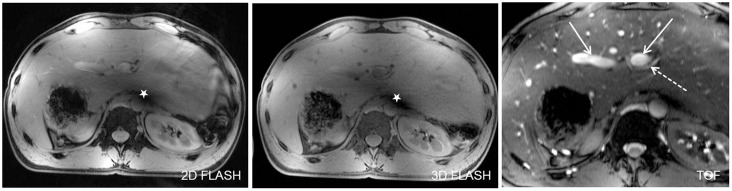
Figure 5 shows an anatomic variability of the upper abdomen in one healthy subject with a tall, centrally arranged liver and partially inverted situs, by means of mirrored location of spleen and stomach in the right upper abdomen. In this subject residual B_1_-artifacts strongly impaired the conspicuity of central liver vessels in 2D and 3D FLASH imaging (stars in Figure A). Yet, being the sequence to be least impaired, TOF imaging allowed for a high-quality assessment of portal vein (arrow) and the left hepatic artery (dashed arrow). Figure B shows high quality delineation of the splenic artery and several small branches at the splenic hilus in all three sequences (arrows).

Analysis of all liver vessel segments demonstrated a significant superiority for TOF imaging, providing a homogeneous hyperintense delineation of all liver vessels (Fig. 5). Mean values of all assessed vessel segments were similar, ranging from 4.13±0.74 for assessment of the common hepatic artery to 4.13±0.54 for the inferior vena cava and 4.21±0.51 for the main portal vein. The right portal vein was the vessel segment to be best delineated with mean score values of 4.63±0.49. Furthermore, this sequence was least impaired by B_1_ inhomogeneity (4.13±0.34) and susceptibility artifacts (4.63±0.49) out of all three sequences (p-value<0.01, respectively) (Fig. 6).

Quantitative analysis confirmed the high quality assessment of the arterial and venous liver vasculature with CR values ranging from 0.43±0.15 (PHA) to 0.59±0.08 (RPV). These CR values were considerably higher in TOF imaging compared to 2D and 3D FLASH imaging (p = 0.02) due to an effective background saturation of liver tissue signal.

Interobserver reliability was calculated moderate for the TOF sequence yielding mean values of 0.45 (image quality) and 0.40 (presence of artifacts).

## Discussion

Noninvasive imaging techniques such as contrast-enhanced computed tomographic and magnetic resonance angiography have emerged to become excellent non-invasive alternatives to conventional catheter angiography for the evaluation of hepatic vasculature [30]–[35]. Gadolinium-based contrast agents for MRA were considered to be safe until first studies characterized Nephrogenic Systemic Fibrosis as a severe systemic side effect associated to the administration of gadolinium-based contrast agents in patients with acute or chronic renal insufficiency [36], [37]. Owing to the onset of this severe side effect, the focus of research has shifted to non-contrast-enhanced MR angiographic techniques for the assessment of hepatic vasculature.

Recent studies at 1.5 Tesla have demonstrated the diagnostic potential of several strategies for liver vessel imaging without the need of contrast media [4], [6], [38]. The most common technique for non-enhanced MR angiography is based on the application of true steady-state free-precession sequences (SSFP).

Within the last few years ultra-high-field MR imaging at 7 Tesla has been introduced to in vivo human imaging. First approaches in neuro imaging revealed an interesting incidental finding, in terms of an inherently high signal intensity of the arterial vasculature in T1w imaging. This incidental finding has been shown beneficial and of high diagnostic value for non-enhanced intracranial MR angiographic applications [16], [17], [27], [39]. With etiology remaining to be incompletely understood, a combination of steady-state and inflow effects as well as the utilization of local transmit/receive coils within a small excitation volume seem to be accountable [40]. Thus, flowing spins are not pre-saturated by RF pulses when entering the imaging region [40].

Based on continuous development of multi-channel body coils and RF shimming technology at 7 T, the focus of research has expanded to whole-body applications, including abdominal imaging. Furthermore, the afore-mentioned hyperintense signal of the arterial vasculature could be confirmed for abdominal vessels [9], [12], [41], [42]. Metzger et al. investigated the feasibility of non-enhanced renal MRA at 7 Tesla utilizing a respiratory triggered turbo-flash sequence [9], [41]. In further 7 T studies, Umutlu et al. demonstrated the feasibility of non-enhanced as well as contrast-enhanced renal MRA. Their results showed promising results of non-enhanced renal arterial vessel imaging, utilizing T1w FLASH and TOF sequences with TOF MRA yielding best ratings in qualitative and quantitative analysis [12].

Our results go in line with previous publications on 7 T renal MRA in demonstrating the feasibility of high-quality T1w non contrast-enhanced imaging of abdominal vasculature at 7 Tesla. In accordance to a previous publication on non-enhanced renal MRA at 7 T by Umutlu et al. [12], our results also confirmed the considerable superiority of TOF MRA, regarding qualitative and quantitative evaluation due to sharply defined and homogeneously hyperintense vessel signal as well as an effective suppression of background signal. 2D and 3D FLASH imaging achieved a similar image quality and clearly lower contrast values with a slight advantage for the 2D FLASH sequence. Nevertheless, out of the three sequences, 3D FLASH imaging provided fastest acquisition (24 seconds) covering the entire liver during one breath-hold and offering high spatial resolution vessel imaging. Yet, in contrast to selective arterial vessel imaging, our results demonstrate the potential of T1w 7 T MRI not only for arterial vessels but for assessment of all three vessel types. TOF MRA revealed best delineation of the right portal vein and equivalently high quality delineation of the main portal vein as well as arterial and venous liver vasculature. Nevertheless, the comparatively long examination time for TOF-MRA has to be mentioned, as five imaging steps of 33 seconds each were necessary to cover the entire liver. Achieving an acquisition time within breath-hold conditions for the rather high flip angle TOF sequence was not possible. Best results regarding image quality and workflow were obtained by using a lower spatial resolution compared to FLASH acquisitions. However, to compensate for the reduced matrix the FOV of the TOF sequence was reduced to a minimum for whole-liver coverage. As an alternative, respiratory-gated imaging using a navigator is still restricted at 7 T due to an inhomogeneous excitation. At last, the breath-hold sequences acquired in the current study are advantageous regarding acquisition time compared to non-enhanced liver MRA studies at lower field strength based on SSFP imaging with acquisition times from 6–10 minutes [4], [5].

Moreover, SSFP imaging is known to suffer from signal heterogeneity of the static magnetic field B_0_ with increasing magnetic field strength, resulting in signal loss or dark banding artifacts [13]. An additional impairment of SSFP sequences can also be caused by SAR limitation, as SAR increases approximately with the square of the magnetic field and with the square of the flip angle. T2w Fast Spin Echo (FSE) imaging has also been proposed for non-enhanced MRA [43]. However, TSE performance at ultra-high field strength is strongly constrained by B_1_ field signal alterations and by a lack of available RF power for generation of accurate refocusing pulses and high flip angles [13]. Hence, as described in previous trials on 7 T abdominal MRI, SSFP and T2w TSE imaging are strictly limited [10], leaving the exploitation of the inherently hyperintense vessel signal in T1 MRI to be a more promising method for non-contrast-enhanced liver vessel imaging at 7 Tesla.

Clearly, our initial study is not without some limitations. With increasing field strength, the Larmor wavelength is reduced to approximately 15 cm at 7 T, provoking significant B_1_-field signal alterations and signal drop outs in large cross-sectional imaging [13]. These artifacts could be reduced in this trial with static RF shimming, pooling the majority of signal voids to a periaortal focus. Nevertheless, these residual B_1_ artifacts caused a predominantly moderate impairment in 2D and 3D FLASH imaging, impeding the delineation of surrounding vessel structures as the main portal vein, the inferior vena cava and the common hepatic artery. Another limitation may be caused by the concomitant delineation of arterial, venous and portal venous vessels. Selective vessel delineation by dedicated vessel signal saturation would have been desirable. Yet, as stated in previous publications on 7 Tesla renal MRA [12], sufficient saturation of the inherently high vessel signal was not applicable, mainly based on B_1_ inhomogeneities, SAR and peak RF power limitations associated with increasing field strength and limited coverage of the local transmit coil in contrast to a whole-body transmit coil used at clinical field strength. For body imaging at 7 T, hardware solutions in addition to improved pulse sequences are necessary. For example, Wu et al. have recently shown improved excitation homogeneity in liver MRI at 7 T with multi-spoke slice-selective parallel transmit RF pulse [44]. Additionally, further improvement of selective vessel signal suppression should be addressed in upcoming trials to improve dedicated vessel diagnostics. Finally, inflow effects depending on differences in slice thickness and TR between the three sequences, and in combination with aforementioned B1 inhomogeneities may have introduced a bias which might partially explain differences in the performance of the three sequences.

In conclusion, the initial results of our study demonstrate the feasibility of non-contrast-enhanced 7 Tesla liver vessel imaging and the challenges of this emerging technique. Non-enhanced T1w imaging in general and TOF MRA in particular, appear to be promising techniques for good quality liver vessel assessment at 7 T. Comparison trials to contrast-enhanced MRA in healthy subjects and patients with liver vessel pathologies should be addressed in future studies to assess the diagnostic competence of non-enhanced liver vessel MRI at 7 T.
